# Increased ATPase activity promotes heat-resistance, high-yield, and high-quality traits in rice by improving energy status

**DOI:** 10.3389/fpls.2022.1035027

**Published:** 2022-12-19

**Authors:** Tingting Chen, Jiaying Ma, Chunmei Xu, Ning Jiang, Guangyan Li, Weimeng Fu, Baohua Feng, Danying Wang, Zhihai Wu, Longxing Tao, Guanfu Fu

**Affiliations:** ^1^ National Key Laboratory of Rice Biology, China National Rice Research Institute, Hangzhou, China; ^2^ Agronomy College, Jilin Agricultural University, Changchun, China; ^3^ Jiangsu Key Laboratory of Crop Genetics and Physiology, Jiangsu Co-Innovation Center for Modern Production Technology of Grain Crops, Agricultural College, Yangzhou University, Yangzhou, China

**Keywords:** rice, heat resistance, yield, quality, energy status, ATPase

## Abstract

Heat stress during the reproductive stage results in major losses in yield and quality, which might be mainly caused by an energy imbalance. However, how energy status affected heat response, yield and quality remains unclear. No relationships were observed among the heat resistance, yield, and quality of the forty-nine early rice cultivars under normal temperature conditions. However, two cultivars, Zhuliangyou30 (ZLY30) and Luliangyou35 (LLY35), differing in heat resistance, yield, and quality were detected. The yield was higher and the chalkiness degree was lower in ZLY30 than in LLY35. Decreases in yields and increases in the chalkiness degree with temperatures were more pronounced in LLY35 than in ZLY30. The accumulation and allocation (ratio of the panicle to the whole plant) of dry matter weight and non-structural carbohydrates were higher in ZLY30 than in LLY35 across all sowing times and temperatures. The accumulation and allocation of dry matter weight and non-structural carbohydrates in panicles were higher in ZLY30 than in LLY35. Similar patterns were observed in the relative expression levels of sucrose unloading related genes *SUT1* and *SUT2* in grains. The ATP content was higher in the grains of LLY35 than in ZLY30, whereas the ATPase activity, which determined the energy status, was significantly lower in the former than in the latter. Thus, increased ATPase activity, which improved the energy status of rice, was the factor mediating the balance among heat-resistance, high-yield, and high-quality traits in rice.

## Introduction

Our planet has warmed by 1.1°C since the late eighteen century, and the warming trend is likely to continue (Keyßer and Lenzen, 2021; Tollefson, 2021). The increase in global mean annual temperature is predicted to reach 1.5°C over the next 20 years if current levels of greenhouse gas emissions are sustained (Michelozzi and De’ Donato, 2021). In such a climate, extreme events, including heat stress, will occur more frequently and decrease crop production (Ferguson et al., 2021; Jagadish et al., 2021). This poses a severe threat to global food security given that the world’s population is projected to reach 9.7 billion by 2050 (United Nations, Department of Economic and Social Affairs, Population Division, 2013; United Nations, Department of Economic and Social Affairs, Population Division, 2015; Bergaglio, 2017). Rice is one of the world’s most important crops, as it feeds half of the world’s population; it is a particularly important staple crop in East and Southeast Asia. However, rice is susceptible to heat stress at the reproductive stage (Espe et al., 2017; Jagadish, 2020; Lu et al., 2021). Heat stress can negatively affect rice yield and quality (Chen et al., 2019; Islam et al., 2019; Jiang et al., 2020; Xu et al., 2020a). There is thus a pressing need to breed high-yield and high-quality rice crops with heat resistance, as this is considered the most effective strategy for maintaining food security in high-temperature climates (Biswal et al., 2019; Yu and Li, 2021). However, cultivars with such properties have not yet been obtained, which we deduced that it might stem from energy deficits or the misallocation of energy in plants under stress conditions.

Adenosine triphosphate (ATP) is the energy currency of living cells required for the energy-consuming processes of growth, motility, and stress responses (De Block and Van Lijsebettens, 2011; Ramon et al., 2019). The tricarboxylic acid cycle and mitochondrial electron transport chain are involved in cellular respiration and ATP production (Millar et al., 2011; Meyer et al., 2019; Fu et al., 2020). Therefore, mitochondrial activity and energy availability affect the control of cell division through the *de novo* biosynthesis of proteins, lipids, nucleic acids, and carbohydrate molecules, as these are energy-demanding processes (Lee and Millar, 2016; Li et al., 2017; He et al., 2018; Vanlerberghe et al., 2020). Several proteins are involved in stress responses and the formation of crop yield and quality. For example, sucrose transporters are required for loading sucrose in source organs, such as leaves, and unloading sucrose into sink organs, such as grains (Wu et al., 2018; Zhang et al., 2018). Grain filling is mainly driven by the activity of sucrose synthase, soluble starch synthase, and starch branching (Yang et al., 2001; Wang et al., 2019). Under heat stress, the activity of antioxidant enzymes, such as superoxide dismutase, peroxidase, and catalase, and the accumulation of heat shock proteins are enhanced (Jacob et al., 2017; Li et al., 2020a; Li et al., 2020b; Ling et al., 2021). Active transport and protein turnover are the two most energy-demanding processes in cells (Scheurwater et al., 2000); these processes are involved in responses to stress and the formation of yield and quality and inevitably compete for energy produced by respiration, especially under limited energy conditions (Neilson et al., 2013; Yang et al., 2021). This explains why high-yield and high-quality cultivars showing heat resistance are scarce. Maximizing ATP production capacity under heat stress conditions is thus a major challenge requiring research attention (De Block and Van Lijsebettens, 2011).

There is an upper bound to the ATP production capacity in cells given that severe stress generally results in slower growth. This suggests that there is competition between growth and maintenance respiration for the limited available energy (Zakrzewska et al., 2011; Caspeta et al., 2014; Amthor et al., 2019). Protein turnover is increased at higher temperatures, and this incurs a high energy cost when energy production or energy utilization efficiency is low (Araújo et al., 2011; Li et al., 2018; Qi et al., 2020); the growth rate is negatively correlated with protein turnover in Arabidopsis plants (Ishihara et al., 2017). Proteins involved in energy production are inactivated by abiotic stress and thus affect the efficiency of ATP production (Yang et al., 2011; Dahal et al., 2014; Li et al., 2020c; Rashid et al., 2020). This can lead to the inactivation of many thermolabile proteins, result in the accumulation of harmful reactive oxygen species, and induce programmed cell death by impairing antioxidant capacity and reducing the accumulation of heat shock proteins (Feng et al., 2018; Jagadish et al., 2021). Abscisic acid can negatively regulate the heat tolerance of rice with rolled leaves by increasing leaf temperature and respiration, which results in the consumption of excess carbohydrates and an energy deficit (Li et al., 2020b). In contrast, acid invertase enhances energy production and reduces energy consumption in the spikelets of rice to prevent the cessation of pollen tube growth induced by heat stress (Jiang et al., 2020). However, the ATP content is higher in the leaves of cold-susceptible cultivars than in cold-resistant cultivars, but the ATPase activity and glutathione content are lower in the former than in the latter (Yu et al., 2020). This indicates that the damage to susceptible cultivars induced by cold stress does not stem from the energy deficit but that the available energy is determined by ATP hydrolysis (Yu et al., 2020). Additionally, a marked increase in energy production efficiency has been observed in rice plants at the vegetative growth phase under moderate high-temperature conditions, but the ATP content and dry matter weight are substantially decreased under such conditions (Li et al., 2021a), suggesting that most of the energy is allocated to maintenance respiration rather than growth respiration (Amthor et al., 2019; Sadok and Jagadish, 2020). This suggests that energy production capacity, as well as energy allocation and utilization, are involved in heat resistance and the formation of rice yield and quality; however, no studies to date have confirmed this hypothesis. Here in this study, we conducted our experiment mainly by using two cultivars ZLY30 and LLY35. The former was characterized by heat resistance, high yield, and high quality, whereas the latter was characterized by heat susceptibility, low yield, and poor quality. We then measured the accumulation and allocation of dry matter weight and non-structural carbohydrates (NSC), the activity of sucrose transporters, the ATP content, and ATPase activity to reveal the mechanisms underlying the trait differences between these cultivars.

## Materials and methods

### Experimental materials and growth conditions

Three experiments were conducted in a paddy field and pots in a plant growth chamber from 2018 to 2020 at the China National Rice Research Institute, Hangzhou, China.

**Experiment I:** Forty-nine early rice cultivars, which were largely planted in the middle and lower reaches of the Yangtze River in China, were grown in a paddy field and pots in 2018. The rice seeds were soaked for 48 h and then sprout at 37°C for 24 h. The rice seeds were sown in a seedling bed on March 25, and seedlings with 3–4 leaves were transplanted to the paddy fields of each plot (10 m^2^) at a hill spacing of 20×20 cm. When mature, rice plants were harvested to determine the yield and quality. Three seedlings were grown in pots (30 cm radius and 30 cm height) filled with 15 kg of paddy soil under normal temperature conditions until flowering. These rice plants were divided into two groups at anthesis; one group of rice plants was subjected to heat stress (38°C from 09:00 am to 04:00 pm and 28°C from 04:01 pm to 8:59 am for 7 d), and the other group served as the control (28°C from 09:00 am to 04:00 pm and 23°C from 04:01 pm to 8:59 am for 7 d). An automatic temperature control system was used to control both temperature and relative humidity. Both groups were maintained at 70–80% relative humidity and natural sunlight conditions. After heat stress ended, rice plants were moved to a net-house until maturity. Twenty cultivars differing in heat resistance and yield were selected to determine quality. Heat resistance was evaluated by calculating the heat stress index (HSI), which was determined using the following formula:


$$\frac{Seed-setting\;rate\;under\;control-Seed-setting\;rate\;under\;heat\;stress\;}{Seed-setting\;rate\;under\;control}$$

**Experiment II:** Ten cultivars differing in heat resistance, yield, and quality were selected based on the data from Experiment I. The experimental design was similar to that of Experiment I; the rice plants were planted in both a paddy field and pots, and the HSI, yield, and quality were determined.

**Experiment III:** A heat-resistant, high-yield, and high-quality cultivar Zhuliangyou 30 (ZLY30) and a heat-susceptible, low-yield, and poor-quality cultivar Luliangyou 35 (LLY35) were selected based on the data from Experiment II and subjected to further experiments. Two cultivars were planted at different sowing dates: March 20, 25, and 30 and April 4 and 9 at a hill spacing of 20×20 cm. Three seedlings were grown in pots (30 cm radius and 30 cm height) filled with 15 kg of paddy soil under normal temperature conditions until flowering. At anthesis, the plants were subjected to different temperatures for 15 d: 28/23°C (day/night), 33/26°C (day/night), and 36/30°C (day/night; day, 09:00 am to 04:00 pm/night, 04:01 pm to 8:59 am). Fresh leaf, sheath, stem, and panicle samples were collected at 15 d after anthesis to determine indicators of sugar and energy metabolism. At maturity, rice plants were sampled to determine the dry matter weight, yield, and quality.

### Determination of dry matter weight, yield, and quality

At maturity, rice plants were divided into leaves, sheath and stems, and panicles and dried at 80°C for 48 h, and the dry matter weight was measured. The panicle number per plant, grain number per panicle, seed-setting rate, and kernel weight were measured to calculate yield. The quality was determined by the Rice Product Quality Inspection Supervision & Testing Center of MOA (Chen et al., 2019).

### Determination of soluble sugar, starch, and NSC content

The sulfuric acid anthrone colorimetric method with some modifications was used to determine the content of soluble sugar and starch (Dubois et al., 1956); the absorbance at 485 nm was measured using a spectrophotometer (Lambda 25, Perkin Elmer, Freemont, CA, USA). Total NSC were determined by calculating the sum of the content of soluble sugars and starch (Jiang et al., 2020).

### ATP content and ATPase activity measurements

An ELISA method and an assay kit were used to measure the ATP content and the ATPase activity according to the manufacturer’s instructions (Shanghai Enzyme-linked Biotechnology Co., Ltd., China). Briefly, 0.1 g of frozen leaves were extracted with 0.1 M PBS (pH 7.4). The homogenate was centrifuged at 3,000 × g for 20 min at 4°C, and the supernatant was collected and determined at 450 nm (Yu et al., 2020).

### Poly-ADP-ribose polymerase (PARP) activity

An assay kit was used to determine the PARP activity by the ELISA method per the manufacturer’s instructions (Shanghai Enzyme-linked Biotechnology Co., Ltd., China). Briefly, approximately 0.2 g of frozen leaves were extracted with 0.1 M PBS (pH 7.4). The homogenate was centrifuged for 20 min at 3,000 × g, and the supernatant was collected and determined at 450 nm (Yu et al., 2020).

### RNA extraction and qRT-PCR analysis

Total RNA was extracted from 100 mg of grains using TRIpure reagent (Aidlab Biotechnologies, Beijing, China). RNA was converted into first-strand cDNA using ReverTra Ace qPCR RT Master Mix (Toyobo, Shanghai, China) with oligo-dT primers. The resultant cDNA was used as a template for quantitative PCR amplification in a Thermal Cycler Dice Real-Time System II (Takara Biotechnology, Dalian, China) with SYBR GreenI(Toyobo, Shanghai, China) as a fluorescent reporter. Primers were designed to generate 150- to 250-bp fragments using PRIMER5 software (Rozen and Skaletsky, 2000). The relative expression levels of nine genes involved in sugar synthesis, transport, and conversion were measured. The primers used for qRT-PCR amplification are listed in Additional file 1: **Table S1**. PCR reactions and detection were carried out following previously described methods. The 2*^−ΔΔCT^* method was used to analyze the relative expression levels of genes, and data were presented as means of triplicate experiments.

### Statistical analysis

SPSS software 11.5 (IBM Corp., Armonk, NY, USA) was used to conduct statistical analyses. The mean values and standard errors in the tables and figures correspond to data from three experimental replicates unless otherwise stated. T-tests and analysis of variance followed by least significant difference test were used to detect significant differences, and the threshold for statistical significance was *p* ≤ 0.05.

## Results

### Heat response, yield, and quality among the forty-nine early rice cultivars

Forty-nine early rice cultivars were planted in pots and subjected to a normal temperature (28°C) and heat stress (38°C) at anthesis. Under normal temperature, the seed-setting rate ranged from 60.0% to 93.8% in these cultivars (**Table 1**). The seed-setting rate was significantly reduced under heat stress compared with control conditions, and the average decrease was 65.0%. The seed-setting rate ranged from 5.0% to 64.3% in these cultivars under heat stress. The yields of cultivars planted in the paddy field ranged from 363.6 kg·666.7 m^-2^ to 549.5 kg·666.7 m^-2^, and the average value was 464.0 kg·666.7 m^-2^. No significant relationships were observed among HSI, the seed-setting rate, and yield in early rice cultivars under normal temperature conditions (**Figure S1**). Under heat stress, there was a significant relationship between the HSI and the seed-setting rate. This suggested that heat resistance was not related to the seed-setting rate and yield under normal temperature conditions. Therefore, 10 heat-resistant cultivars (low HSI) with high yields as well as 10 heat-susceptible cultivars (high HSI) with low yields were selected for quality determination (**Table S2**). The average value of quality parameters varied little among these two groups of cultivars (**Table S3**). However, the brown rice rate, milled rice rate, grain length, aspect ratio, alkali spreading value, gel consistency (GC), and amylose content (AC) were higher in heat-resistant cultivars than in heat-susceptible cultivars, and the head rice rate (HRR), chalkiness degree (CD), and brown rice protein content were lower in heat-resistant cultivars than in heat-susceptible cultivars (**Table S3**). There were no differences in yield, HSI, and quality among heat-resistant and heat-susceptible cultivars (**Tables S2**, **S3**). This indicates that variation in heat resistance, yield, and quality was greater among individuals than between groups and thus that only certain individuals possessed heat resistance, coupled with high yield and quality.

**Table 1:** Heat resistance and yield varied among forty-nine early rice cultivars.

Cultivars	Seed-setting rate (%)		Heat stress index	Grain yield (kg/666.7m^2^)
	Control	Heat stress		
**Yiliangyou 4156**	88.5 ± 7.0	47.6 ± 4.7	0.462	549.5 ± 33.0
**Lingliangyou 722**	84.3 ± 7.4	20.8 ± 5.0	0.753	538.4 ± 15.9
**Wuyou 463**	79.0 ± 9.8	36.1 ± 6.1	0.543	534.6 ± 35.6
**Zhongjiazao 17**	92.0 ± 3.0	30.6 ± 5.5	0.667	533.3 ± 63.3
**Liangyouzao 17**	79.7 ± 5.5	43.7 ± 7.0	0.452	526.9 ± 32.4
**Zaoxian 009**	89.7 ± 4.4	25.2 ± 4.7	0.719	526.7 ± 19.0
**Jinyou 458**	77.0 ± 7.5	45.0 ± 21.3	0.416	515.4 ± 53.4
**Lingliangyou 32**	85.2 ± 5.7	18.4 ± 3.3	0.784	513.0 ± 53.0
**Rongyou 585**	82.4 ± 10.2	64.3 ± 8.0	0.220	507.6 ± 17.6
**You I336**	76.5 ± 7.8	55.9 ± 5.3	0.270	505.5 ± 46.7
**Zaofengyou 402**	77.8 ± 6.5	32.2 ± 5.4	0.586	504.6 ± 34.2
**Changliangyou 35**	73.7 ± 10.1	14.8 ± 3.1	0.800	501.7 ± 22.1
**Qiliangyou 2012**	88.0 ± 6.3	8.1 ± 2.8	0.908	491.4 ± 35.7
**Zhongzu 7**	87.1 ± 3.3	21.7 ± 3.7	0.751	487.4 ± 19.8
**Wen 814**	93.8 ± 3.1	11.1 ± 1.9	0.881	484.1 ± 20.3
**Yongxian 975**	92.8 ± 1.1	5.0 ± 1.0	0.946	483.4 ± 21.7
**Xinliangyou 2045**	85.8 ± 2.6	20.2 ± 2.9	0.765	480.4 ± 24.5
**Lingliangyou 7421**	85.6 ± 7.6	15.1 ± 3.5	0.823	478.7 ± 23.4
**Wufengyou 286**	75.0 ± 8.5	21.6 ± 4.1	0.712	478.1 ± 15.9
**Xiangzaoxian 24**	90.3 ± 4.7	26.5 ± 4.7	0.707	476.4 ± 26.6
**Xiannong 25**	74.5 ± 19.2	34.7 ± 3.9	0.535	474.4 ± 44.1
**Jiangzao 361**	81.8 ± 6.7	17.5 ± 3.0	0.786	473.0 ± 4.4
**Lingliangyou 211**	69.2 ± 8.5	51.6 ± 7.8	0.254	465.8 ± 23.9
**Lingliangyou 942**	64.4 ± 6.2	58.6 ± 7.2	0.090	463.9 ± 24.6
**Zhuliangyou 101**	89.4 ± 6.5	36.8 ± 6.8	0.588	462.8 ± 20.9
**Luliangyou 4026**	83.0 ± 6.4	12.9 ± 3.4	0.844	461.9 ± 40.9
**Zhuliangyou 829**	61.2 ± 6.5	33.4 ± 5.5	0.454	461.9 ± 9.1
**Zhuliangyou 1**	65.7 ± 8.5	7.1 ± 1.8	0.891	459.6 ± 41.5
**Zhuliangyou 609**	60.0 ± 6.0	36.8 ± 8.6	0.386	459.6 ± 66.2
**Lingliangyou 396**	82.5 ± 6.9	16.3 ± 2.1	0.803	458.5 ± 8.9
**Zhuliangyou 30**	76.4 ± 6.9	38.9 ± 7.0	0.491	454.0 ± 21.8
**Zhongleng 23**	71.0 ± 9.8	39.7 ± 5.1	0.441	450.4 ± 16.7
**Zhuliangyou 39**	63.5 ± 8.8	13.1 ± 3.1	0.794	441.9 ± 39.4
**Tanliangyou 83**	92.9 ± 2.9	47.6 ± 6.8	0.487	440.0 ± 10.3
**Xiangzaoxian 42**	90.2 ± 4.6	13.4 ± 3.5	0.851	438.1 ± 61.5
**Zhongzao 39**	85.9 ± 4.6	5.8 ± 1.9	0.933	436.7 ± 12.5
**Lingliangyou 7717**	89.2 ± 11.5	13.2 ± 2.8	0.852	436.6 ± 18.7
**Xiangzaoxian 32**	86.9 ± 6.8	24.3 ± 2.7	0.720	435.9 ± 28.2
**Yuenuo 06**	68.4 ± 4.4	11.5 ± 2.7	0.832	433.1 ± 24.2
**Zhuliangyou 4024**	74.3 ± 9.1	42.5 ± 6.7	0.427	432.4 ± 18.6
**Xiangzaoxian 45**	86.2 ± 5.7	35.2 ± 5.8	0.592	418.5 ± 17.5
**Zhongzao 35**	79.7 ± 5.2	23.0 ± 5.1	0.712	417.9 ± 64.5
**Ronryou 286**	87.6 ± 5.8	25.6 ± 3.4	0.746	417.5 ± 15.4
**Luliangyou 35**	81.3 ± 10.5	6.6 ± 2.1	0.919	410.6 ± 25.0
**Lingliangyou 611**	70.2 ± 9.6	9.6 ± 1.4	0.863	409.2 ± 43.6
**Zhuliangyou171**	66.1 ± 9.1	37.2 ± 4.8	0.437	404.9 ± 3.4
**Zhuliangyou 312**	82.2 ± 6.1	50.7 ± 6.8	0.384	403.1 ± 51.7
**Xiangzaoxian 6**	85.1 ± 4.7	5.6 ± 1.9	0.934	401.0 ± 30.6
**Ganzaoxian 51**	81.8 ± 3.3	10.4 ± 1.9	0.873	363.6 ± 21.8

### Further selection of rice cultivars differing in traits of heat tolerance, grain yield and rice quality

To select cultivars with heat resistance, high yield, and high quality, 10 cultivars varying in heat resistance and yield were selected according to the data collected above and planted in 2019 (**Table 2**). The HSI of ZLY30, Liangyouzao17, Lingliangyou 942, Yiliangyou 4156, and Zhuliangyou 829 was significantly lower than that of Rongliangyou 286, Zhongzao 35, Lingliangyou 611, Zhongzao 39, and LLY35, and yield was higher in the former set of cultivars than in the latter set. The lowest HSI was observed in ZLY30, and the highest HSI was observed in LLY 35. The highest yield was observed in Liangyouzao 17, followed by ZLY30, and the lowest yield was observed in Rongliangyou 286 and Zhongzao 39. Little variation in quality was observed between these two groups of rice cultivars except for HRR and GC. However, higher values of HRR and GC were observed in heat-susceptible rice cultivars with low yield than in heat-resistant cultivars with high yield. No differences were observed among cultivars in HSI, yield, or quality parameters (**Tables 2**, **3**). The HRR, alkali spreading value, grain length, and aspect ratio were higher in ZLY30 than in LLY35, and the CD, chalky grain percentage, GC, and AC were lower in the former than in the latter. Therefore, ZLY30 was considered a heat-resistant, high-yield, and high-quality cultivar, whereas LLY35 was considered a heat-susceptible, low-yield, and poor-quality cultivar; these two cultivars were then examined to characterize the mechanism underlying these trait differences.

**Table 2:** The seed-setting rate, heat stress index and yields vary among 10 early indicia rice varieties with different heat tolerance and yield.

Cultivars	Seed-setting rate (%)		Heat stress index	Grain yield (kg/666.7m^2^)
	Control	Heat stress		
**Zhuliangyou30**	69.8 ± 7.4 cd	49.5 ± 5.6 a	0.291	509.1 ± 10.1 a
**Liangyouzao17**	65.0 ± 1.9 d	42.8 ± 5.1 a	0.342	532.2 ± 27.3 a
**Lingliangyou 942**	85.3 ± 2.7 ab	51.4 ± 6.6 a	0.397	488.1 ± 15.4 ab
**Yiliangyou 4156**	81.3 ± 1.0 b	45.7 ± 7.9 a	0.437	487.6 ± 18.6 ab
**Zhuliangyou 829**	87.0 ± 6.4 ab	48.3 ± 2.3 a	0.445	481.1 ± 38.8 abc
**Rongliangyou 286**	68.7 ± 4.1 cd	30.4 ± 5.6 b	0.557	387.4 ± 20.8 d
**Zhongzao 35**	78.3 ± 8.2 bc	32.6 ± 2.2 b	0.584	472.1 ± 27.6 abc
**Lingliangyou 611**	75.8 ± 7.4 bc	28.5 ± 6.0 b	0.624	475.1 ± 23.7 abc
**Zhongzao 39**	88.3 ± 1.3 a	30.3 ± 5.1 b	0.657	426.1 ± 34.0 cd
**Luliangyou 35**	82.1 ± 6.6 abc	27.8 ± 2.7 b	0.662	466.7 ± 10.3 bc

Data are expressed as mean ± SE (n=6) and different letters in a column indicate statistical significance at the P = 0.05 level among cultivars.

**Table 3:** The quality were showed in the 10 early indica rice varieties with different heat tolerance and yields.

Cultivars	BRR	HRR	MRR	CD	CGP	Translucency	ASV	GC	AC	GL	AR
**Zhuliangyou30**	80.8 ± 0.2 a	8.8 ± 0.1 e	69.0 ± 0.2 a	9.4 ± 0.5 b	48 ± 2.4 b	3	5.8 ± 0.5 a	36 ± 0.6 e	21.6 ± 0.1 c	7.0 ± 0.2 a	3.2
**Liangyouzao17**	80.3 ± 0.1 a	12.4 ± 0.2 b	69.6 ± 0.5 a	16.7 ± 3.2 a	66 ± 4.3 a	3	5.0 ± 0.3 ab	56 ± 0.5 b	22.7 ± 0.2 b	5.9 ± 0.1 b	2.5
**Lingliangyou 942**	78.5 ± 0.2 a	14.6 ± 0.2 a	67.4 ± 0.2 a	3.3 ± 0.2 c	18 ± 1.4 c	2	4.6 ± 0.2 bc	82 ± 2.4 a	23.2 ± 0.2 b	6.6 ± 0.2 a	3.0
**Yiliangyou 4156**	79.6 ± 0.3 a	10.6 ± 0.2 d	67.6 ± 0.4 a	25.8 ± 6.2 a	65 ± 2.4 a	4	4.4 ± 0.2 c	38 ± 0.5 d	19.8 ± 0.2 d	6.0 ± 0.2 b	2.5
**Zhuliangyou 829**	80.9 ± 0.3 a	11.6 ± 0.3 c	70.1 ± 0.3 a	20.0 ± 4.2 a	63 ± 4.5 a	3	5.2 ± 0.4 ab	43 ± 0.8 c	26.5 ± 0.3 a	6.0 ± 0.2 b	2.6
**Average**	80.0 ± 1.0	11.6 ± 2.2	68.7 ± 1.2	15.0 ± 13.2	52.0 ± 20.4	3.0 ± 0.7	5.0 ± 0.6	51.0 ± 19	22.8 ± 2.5	6.3 ± 0.5	2.8 ± 0.3
**CV**	0.011	0.17	0.016	0.53	0.35	0.21	0.098	0.33	0.097	0.068	0.11
**Rongliangyou 286**	80.0 ± 0.33 a	23.1 ± 0.42 a	70.1 ± 0.31 a	0.4 ± 0.08 c	6 ± 0.73 c	1	5.8 ± 0.3 a	46 ± 0.8 d	22.8 ± 0.2 b	6.6 ± 0.1 a	3.0
**Zhongzao 35**	80.0 ± 0.27 a	19.3 ± 0.36 c	67.5 ± 0.36 a	26.7 ± 4.8 a	90 ± 5.28 a	4	5.2 ± 0.2 ab	65 ± 1.3 b	26.6 ± 0.3 a	5.6 ± 0.3 b	2.2
**Lingliangyou 611**	78.8 ± 0.15 a	17.9 ± 0.40 d	69.0 ± 0.71 a	1.9 ± 0.02 b	9 ± 0.13 d	3	3.2 ± 0.2 c	76 ± 3.3 a	13.7 ± 0.2 c	6.3 ± 0.3 a	2.9
**Zhongzao 39**	79.5 ± 0.18 a	20.5 ± 0.54 b	69.9 ± 0.65 a	22.3 ± 3.5 a	71 ± 3.36 b	3	5.0 ± 0.2 b	38 ± 0.8 e	26.9 ± 0.2 a	5.3 ± 0.2 b	2.0
**Luliangyou 35**	80.0 ± 0.38 a	5.1 ± 0.13 e	68.4 ± 0.28 a	26.2 ± 5.6 a	71 ± 4.27 b	3	5.2 ± 0.3 ab	54 ± 1.2 c	26.4 ± 0.4 a	6.2 ± 0.3 a	2.6
**Average**	79.7 ± 0.53	17.2 ± 7.0	69.0 ± 1.1	15.5 ± 13.2	49.4 ± 39.1	2.8 ± 1.1	4.9 ± 1.0	55.8 ± 15.1	23.3 ± 5.6	6.0 ± 0.5	2.5 ± 0.4
**CV**	0.006	0.37	0.014	0.76	0.71	0.35	0.18	0.24	0.22	0.08	0.15

Data are expressed as mean ± SE (n=6) and different letters in a separated column indicate statistical significance at the P = 0.05 level among five cultivars. BRR, Brown rice rate; HRR, Head rice rate; MRR, Milled Rice Ratio; CD, Chalkiness degree; CGP, Chalky grain percentage; ASV, Alkali spreading value; GC, Gel consistency; AC, Amylose content; GL, Grain length; AR, Aspect ratio.

### Yield varied with sowing time and temperature

**Figure 1:**
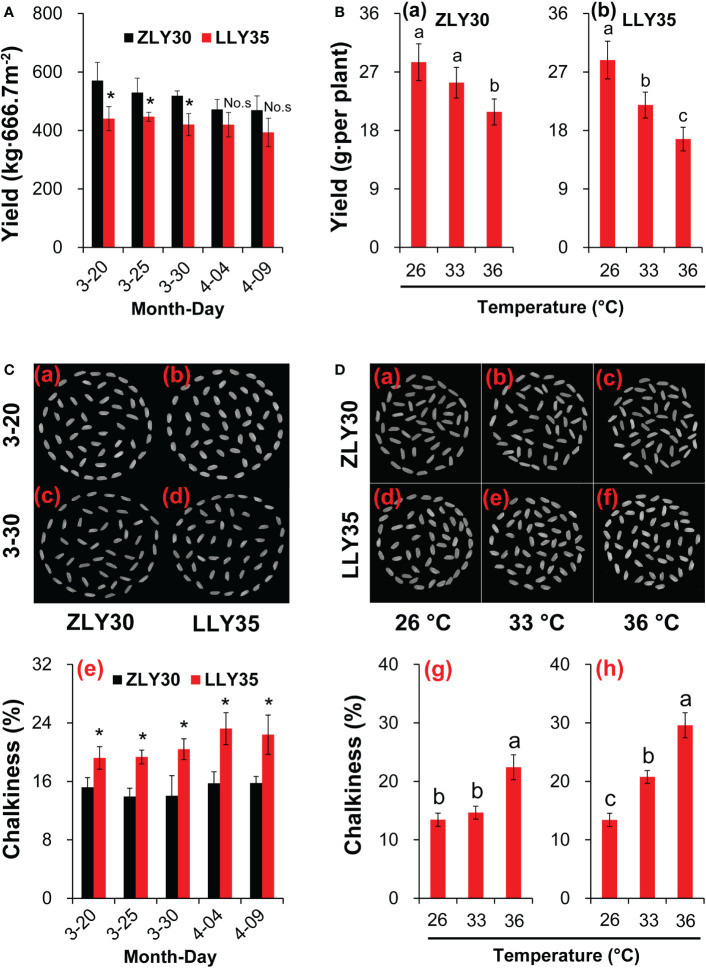
Effect of different sowing times and temperatures on yield and chalkiness degree in grains of rice. **(A)** Yields varied among the sowing times of ZLY30 and LLY35; **(B)** Changes in yields of two cultivars under different temperatures; **(C)** Chalkiness degree varied among the sowing times; **(D)** Changes in chalkiness degree of grains under different temperatures. Vertical bars denote standard deviations (*n*=4). “^*^” indicated significant difference while “No.s” indicate no significant difference at the 0.05 probability level between the ZLY30 and LLY35 within one sowing time using T-test analysis. Different letters indicate significant differences with a least significant difference test at *P ≤* 0.05 among different temperatures within one genotype using one-way analysis of variance.

To study the changes in yield under different environmental conditions, ZLY30 and LLY35 were planted at different sowing times and subjected to different temperature conditions. The yields of the two cultivars varied with sowing time and temperature (**Figures 1A, B**). The yields of ZLY30 were higher than those of LLY35 and were approximately 29.6%, 19.1%, 25.6%, 5.6%, and 11.7% higher in ZLY30 than in LLY35 for sowing dates of March 20, 25, and 30 and April 4 and 9, respectively (**Figure 1A**). However, differences in yield were only significant for sowing dates of March 20, 25 and 30. Yields decreased with temperature (**Figure 1B**). In ZLY30, the yield per pot was 28.5, 25.4, and 20.9 g in the 26, 33, and 36°C treatments, respectively. There was no difference in yield between the 26°C and 33°C treatments, but the yields were significantly higher in these treatments compared with the 36°C treatment. In LLY35, the yield per pot was 28.8, 21.9, and 16.7 g in the 26, 33, and 36°C treatments, respectively. The differences in the yield of LLY35 among treatments were significant.

### CD varies with sowing time and temperature

Chalky degree is an important quality characteristic in the rice grain. It occurs most commonly when grains are exposed to high temperatures during development by affecting starch granules array. Rice quality would deteriorate by appearing obvious opaque area. The CD was significantly higher in LLY35 than in ZLY30 grown in a paddy field across all environmental conditions (**Tables 3**, **S3**). Thus, the CD was determined under different sowing times and temperature conditions (**Figures 1C, D**). The CD was 26.4%, 38.8%, 45.4%, 47.6%, and 42.0% higher in LLY35 than in ZLY30 at sowing dates of March 20, 25, and 30 and April 4 and 9, respectively, and differences between these two cultivars at all sowing dates were significant (**Figure 1C**). The CD increased with temperature in the two rice cultivars (**Figure 1D**). In ZLY30, the CD was 9.0% and 66.7% higher in the 33°C and 36°C treatments compared with the 26°C treatment, respectively; in LLY35, the CD was 54.8% and 120.7% higher in the 33°C and 36°C treatments compared with the 26°C treatment, respectively.

### Dry matter weight and carbohydrate accumulation and allocation under different sowing times and temperature conditions

**Figure 2:**
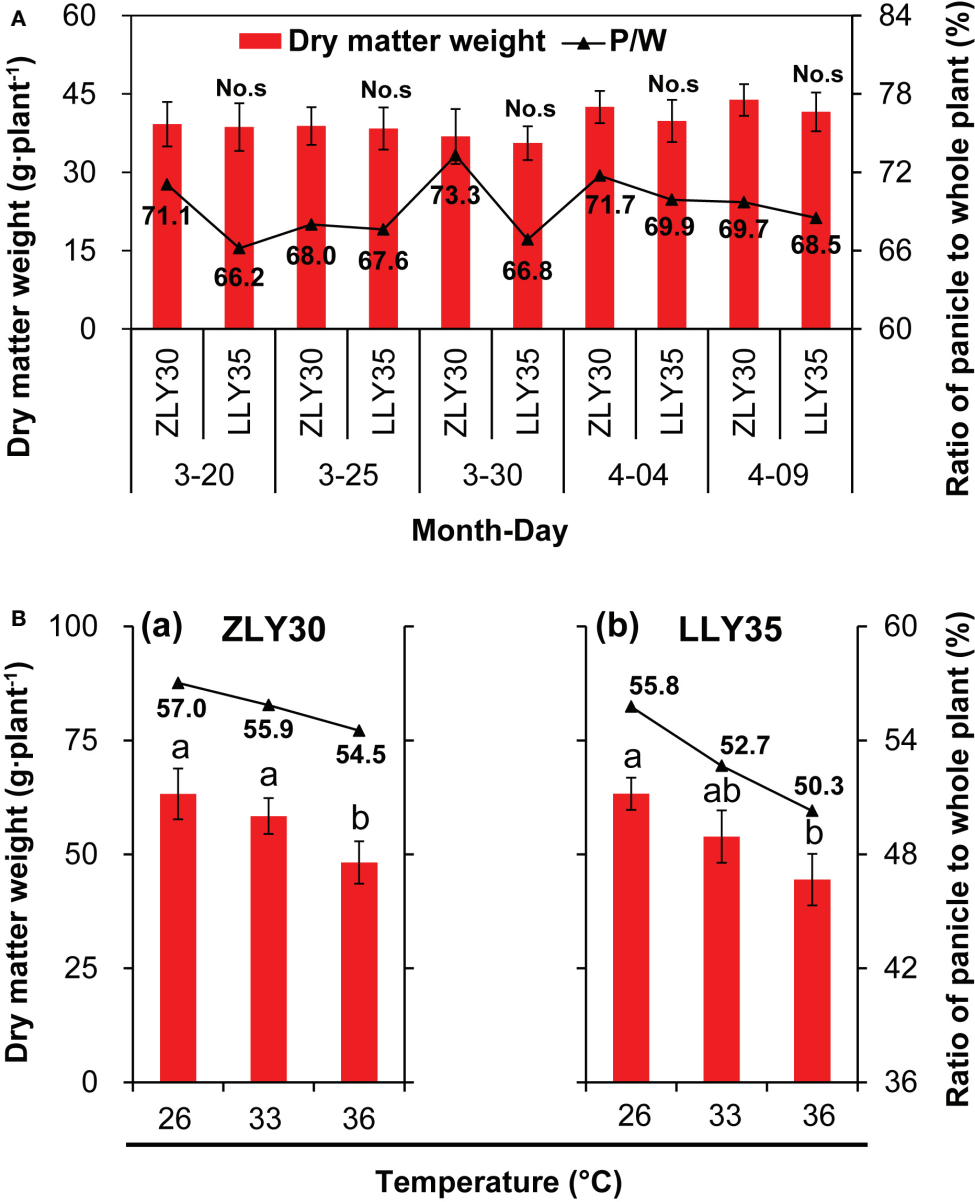
Effect of different sowing time and temperatures on the dry matter weight accumulation and allocation of rice. **(A)** Dry matter weight accumulation and allocation of ZLY30 and LLY35 varied among the sowing times; **(B)** Changes in dry matter weight accumulation and allocation of two cultivars under different temperatures; Vertical bars denote standard deviations (*n*=4). “No.s” indicate no significant difference at the 0.05 probability level between the ZLY30 and LLY35 within one sowing time using T-test analysis. Different letters indicate significant differences with a least significant difference test at *P ≤* 0.05 among different temperatures within one genotype using one-way analysis of variance.

The accumulation and distribution of assimilates is an important factor determining the yield and quality of rice; thus, the dry matter weight and NSC were measured. The dry matter weight of the whole plant, including the leaf, sheath and stem, and the panicle, was higher in ZLY30 than in LLY35 across all sowing dates; however, none of the differences in dry matter weight between cultivars were significant (**Figure 2A**). The ratio of the dry matter weight of the panicle to the whole plant was higher in ZLY30 than in LLY35, especially for sowing dates of March 20 and 30. This ratio was approximately 71.1% and 73.1% for ZLY30 and 66.2% and 66.8% for LLY350 for sowing dates of March 20 and 30, respectively. The dry matter weight of whole plants decreased with temperature in both cultivars (**Figure 2B**). In ZLY30, the dry matter weight was 9.7% and 27.2% lower under 33°Cand 36°Cthan under 26°C, respectively; in LLY35, the dry matter weight was 19.8% and 37.1% lower under 33°C and 36°Cthan under 26°C, respectively. The ratio of the dry matter weight of the panicle to the whole plant decreased with temperature, and this ratio was higher in LLY35 than in ZLY30.

**Figure 3:**
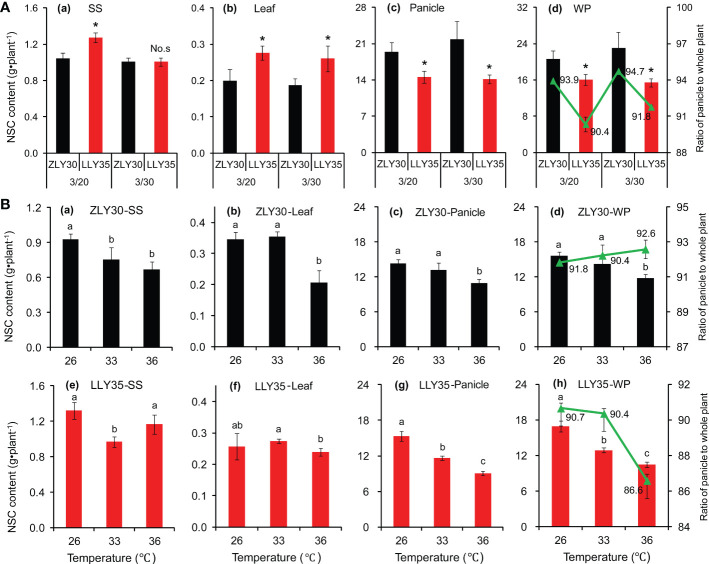
Effect of different sowing times and temperatures on non-structural carbohydrate (NSC) accumulation and distribution of rice. **(A)** NSC accumulation in stem and sheath (SS), leaves (L), panicle (P), whole plant (W) and distribution in ZLY30 and LLY35 at different sowing times; **(B)** Changes in NSC accumulation and allocation of two cultivars under different temperatures; Vertical bars denote standard deviations (*n*=3). “^*^” indicated significant difference while “No.s” indicate no significant difference at the 0.05 probability level between the ZLY30 and LLY35 within one sowing time using T-test analysis. Different letters indicate significant differences with a least significant difference test at *P ≤* 0.05 among different temperatures within one genotype using one-way analysis of variance.

Similar patterns were observed in NSC between the two cultivars under different sowing times and temperature conditions (**Figure 3**). NSC in whole plants were 28.5% and 49.4% higher in ZLY30 than in LLY35 for sowing dates of March 20 and 30, respectively (**Figure 3A**). NSC in the panicle were 33.6% and 54.3% higher in ZLY30 than in LLY35 for sowing dates of March 20 and 30, respectively. The ratio of NSC of the panicle to the whole plant was significantly higher in ZLY30 than in LLY35 for sowing dates of March 20 and 30, respectively. The NSC of the panicle and whole plant decreased with temperature in the two cultivars, and the decrease was more pronounced in ZLY30 than in LLY35 (**Figure 3B**). The ratio of NSC in the panicle to the whole plant increased slightly with temperature in ZLY30 but decreased with temperature in LLY35; this ratio was significantly lower in the 36°C treatment than in the 26°C treatment in LLY35.

### Sucrose transport under different sowing times and temperature conditions

**Figure 4:**
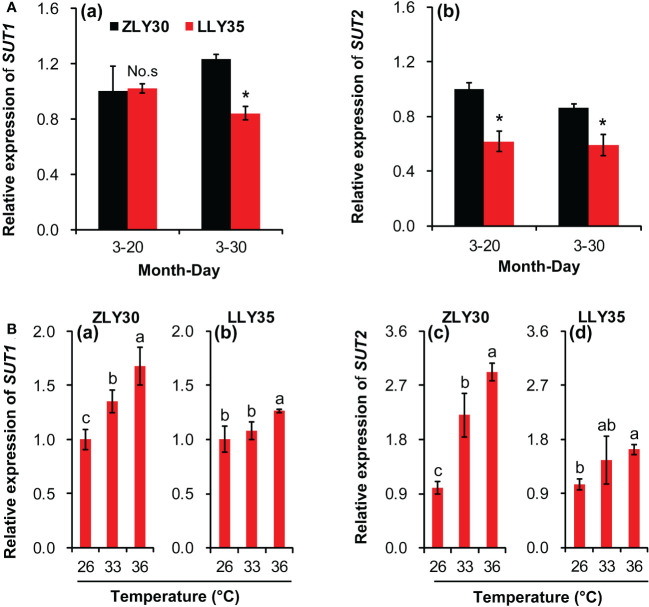
Effect of different sowing times and temperatures on relative expression levels of *SUT1* and *SUT2* in grains of rice. **(A)** Relative gene expression levels of *SUT1* **(a)** and *SUT2* **(b)** in ZLY30 and LLY35 at different sowing times; **(B)** Relative gene expression levels of of *SUT1* **(a, b)** and *SUT2* **(c, d)** in two cultivars under different temperatures; Vertical bars denote standard deviations (*n*=3). “^*^” indicated significant difference while “No.s” indicate no significant difference at the 0.05 probability level between the ZLY30 and LLY35 within one sowing time using T-test analysis. Different letters indicate significant differences with a least significant difference test at *P ≤* 0.05 among different temperatures within one genotype using one-way analysis of variance.

The sucrose transporters (SUT) play key roles in the loading of sucrose in source organs, such as leaves, and the unloading of sucrose in sink organs, such as grains. The relative expression levels of *SUT1* and *SUT2* were determined in grains (**Figure 4**). There was no difference in the relative expression levels of *SUT1* in ZLY30 and LLY35 when they were sown on March 20; however, the expression of these two genes was significantly higher in the former than in the latter when these cultivars were sown on March 30 (**Figure 4A** **, a**). The expression of *SUT2* was markedly increased in ZLY30 compared with LLY35 for sowing dates of March 20 and 30 (**Figure 4A** **, b**). The relative expression levels of *SUT1* and *SUT2* increased with temperature in both cultivars (**Figure 4B**). However, increases in the expression of these two genes with temperature were more pronounced in ZLY30 than in LLY35. Approximately 35.1% and 67.4% increases in the relative expression levels of *SUT1* were observed in ZLY30 under the 33°C and 36°C treatments compared with the 26°C treatment, respectively (**Figure 4B** **, a**); in LLY35, 7.6% and 25.9% increases in the relative expression levels of *SUT1* were observed under the 33°C and 36°C treatments compared with the 26°C treatment, respectively. Approximately 120.5% and 191.5% increases in the relative expression levels of *SUT2* were observed in ZLY30 under the 33°C and 36°C treatments compared with the 26°C treatment, respectively; in LLY35, 38.8% and 55.8% increases in the relative expression levels of *SUT2* were observed under the 33°C and 36°C treatments compared with the 26°C treatment, respectively (**Figures 4B** **, b**).

### Energy status in grains under different sowing times and temperature conditions

**Figure 5:**
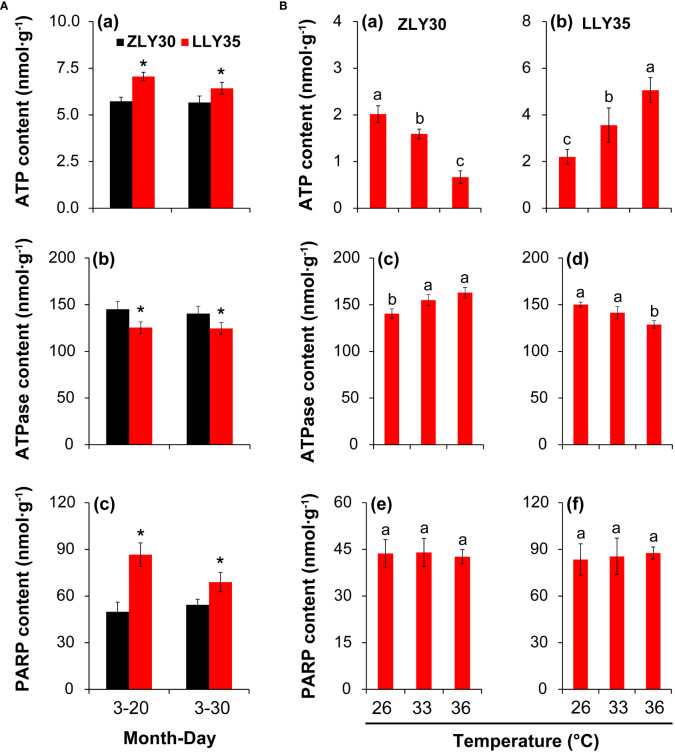
Effect of different sowing times and temperatures on energy metabolism in grains of rice. **(A)**, Content of ATP **(a)**, ATPase **(b)**, and PARP **(c)** in ZLY30 and LLY35 at different sowing times; **(B)**, Content of ATP **(a, b)**, ATPase (c and d), and PARP (e and f) of two cultivars under different temperatures; Vertical bars denote standard deviations (*n*=3). “^*^” indicated significant difference while “No.s” indicate no significant difference at the 0.05 probability level between the ZLY30 and LLY35 within one sowing time using T-test analysis. Different letters indicate significant differences with a least significant difference test at *P ≤* 0.05 among different temperatures within one genotype using one-way analysis of variance.

The energy status is involved in the formation of yield, quality, and heat resistance in plants under different environmental conditions. Therefore, the ATP content, ATPase activity, and PARP activity were determined in grains (**Figure 5**). The ATP content was 18.9% and 11.9% lower in the grains of ZLY30 than in the grains of LLY35 for sowing dates of March 20 and 30, respectively, and these differences were significant (**Figures 5A, a**). The ATPase activity was increased by 13.6% and 11.4% in ZLY30 compared with LLY35 for sowing dates of March 20 and 30, respectively (**Figures 5A** **, b**). The PARP activity was increased by 42.4% and 21.3% in LLY35 compared with ZLY30 for sowing dates of March 20 and 30, respectively (**Figures 5A** **, c**).

The ATP content significantly decreased with temperature in ZLY30 but increased with temperature in LLY35 (**Figures 5B** **, a, b**). The ATP content was 21.4% and 67.2% lower in ZLY30 under the 33°C and 36°C treatments compared with the 26°C treatment, respectively; in LLY35, the ATP content was 62.0% and 130.2% higher under the 33°C and 36°C treatments compared with the 26°C treatment, respectively. The ATPase activity increased with temperature in ZLY30 but decreased with temperature in LLY35 (**Figures 5B** **, c, d**). The ATPase activity was 10.3% and 16.0% higher in ZLY 30 under the 33°C and 36°C treatments compared with the 26°C treatment, respectively; in LLY35, the ATPase activity was 5.8% and 14.3% lower under the 33°Cand 36°C treatments compared with the 26°C treatment, respectively. No significant differences in PARP activity were observed among the three treatments in the two cultivars (**Figures 5B** **, e, f**).

### Energy allocation in grains under different sowing times and temperature conditions

**Figure 6:**
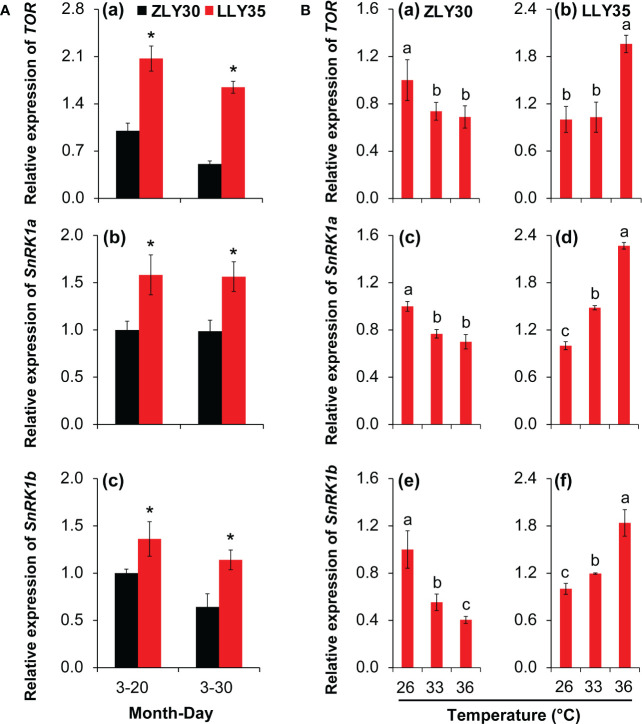
Effect of different sowing times and temperatures on energy allocations in grains of rice. **(A)**, Relative expression of *TOR* **(a)**, *SnRK1a* **(b)**, and *SnRK1b* **(c)** in ZLY30 and LLY35 at different sowing times; **(B)** Relative expression of *TOR* **(a, b)**, *SnRK1a* **(c, d)**, and *SnRK1b* **(e, f)** of two cultivars under different temperatures; Vertical bars denote standard deviations (*n*=3). “^*^” indicated significant difference while “No.s” indicate no significant difference at the 0.05 probability level between the ZLY30 and LLY35 within one sowing time using T-test analysis. Different letters indicate significant differences with a least significant difference test at *P ≤* 0.05 among different temperatures within one genotype using one-way analysis of variance.

The sucrose non-fermenting related protein kinase 1 (SnRK1) and target of rapamycin (TOR) pathways play important roles in energy allocation in plants; thus, the expression levels of *SnRK1a*, *SnRK1b*, and *TOR* were determined in grains (**Figure 6**). Marked increases in the relative expression levels of *SnRK1a*, *SnRK1b*, and *TOR* were observed in LLY35 compared with ZLY30 for sowing dates of March 20 and 30 (**Figures 6A** **, a–c**). The expression levels of *SnRK1a*, *SnRK1b*, and *TOR* decreased with temperature in ZLY30 and increased with temperature in LLY35 (**Figure 6B** **, a–f**).

## Discussion

### Relationships among heat resistance, yield, and quality

Heat stress at the reproductive stage of rice results in yield loss and poor quality, including increases in spikelet sterility and the CD and decreases in the HRR (Nevame et al., 2018; Begcy et al., 2019; Xu et al., 2020; Impa et al., 2021). These findings are consistent with the results of experiments conducted in this study, in which rice plants were subjected to high-temperature conditions (**Figure 1**). Yields were higher and CD was lower in ZLY30 than in LLY35, regardless of sowing time and temperature conditions (**Figure 1** and **Tables 1**-**3**). This suggests that breeding cultivars with heat resistance, high yield, and high quality is the best strategy for maintaining current levels of rice production in high-temperature climates. The development of “smart crops” showing high resistance to extreme weather conditions, as well as high adaptability, high yield, and high quality, is required for addressing the challenges posed by global warming (Yu and Li, 2021). Greater and more consistent crop production will be required against a backdrop of climatic stress that limits yields, owing to shifts in pests and pathogens, precipitation, heat waves, and other weather extremes (Bailey-Serres et al., 2019). However, rice cultivars with such properties are scarce.

No correlations were observed among the yield, quality, and heat resistance of early rice cultivars (**Figure S1**; **Table S4**). This was partly consistent with the results of previous studies examining rice cultivars under heat conditions (Fu et al., 2012). Rice cultivars with high heat resistance often have lower yield or quality under normal temperature conditions (Zhang et al., 2015). However, the transcription factor Ideal Plant Architecture 1 can increase both yield and disease resistance by sustaining a balance between growth and immunity (Wang et al., 2018; Zhang et al., 2020). Recently, the Ca^2+^-sensor RESISTANCE OF RICE TO DISEASES1 was shown to enhance both disease resistance and yield (Gao et al., 2021). Several genes have been reported to confer heat tolerance in rice without compromising yield (Li et al., 2015; Shen et al., 2015; Chen et al., 2020). However, whether these genes can improve quality in addition to improving resistance and yield remains unclear. Extensive bioinformatic analyses of yield components, resistance to rice blast, and taste quality have been conducted, and several alleles for these traits have been identified, which has led to the development of two elite lines, XY99 and JXY1, showing excellent taste, high yield, and broad-spectrum blast resistance (Xiao et al., 2021). Similarly, heat resistance, yield, and quality (low CD) were higher in ZLY30 compared with LLY35, but the variation in AC content between these two cultivars exhibited a different pattern during 2019–2020 (**Tables S2**, **S3**), suggesting that additional research is required to clarify the mechanisms underlying observed changes, including the role of energy metabolism in shaping these traits.

### The role of energy status in affecting heat resistance, yield, and quality

**Figure 7:**
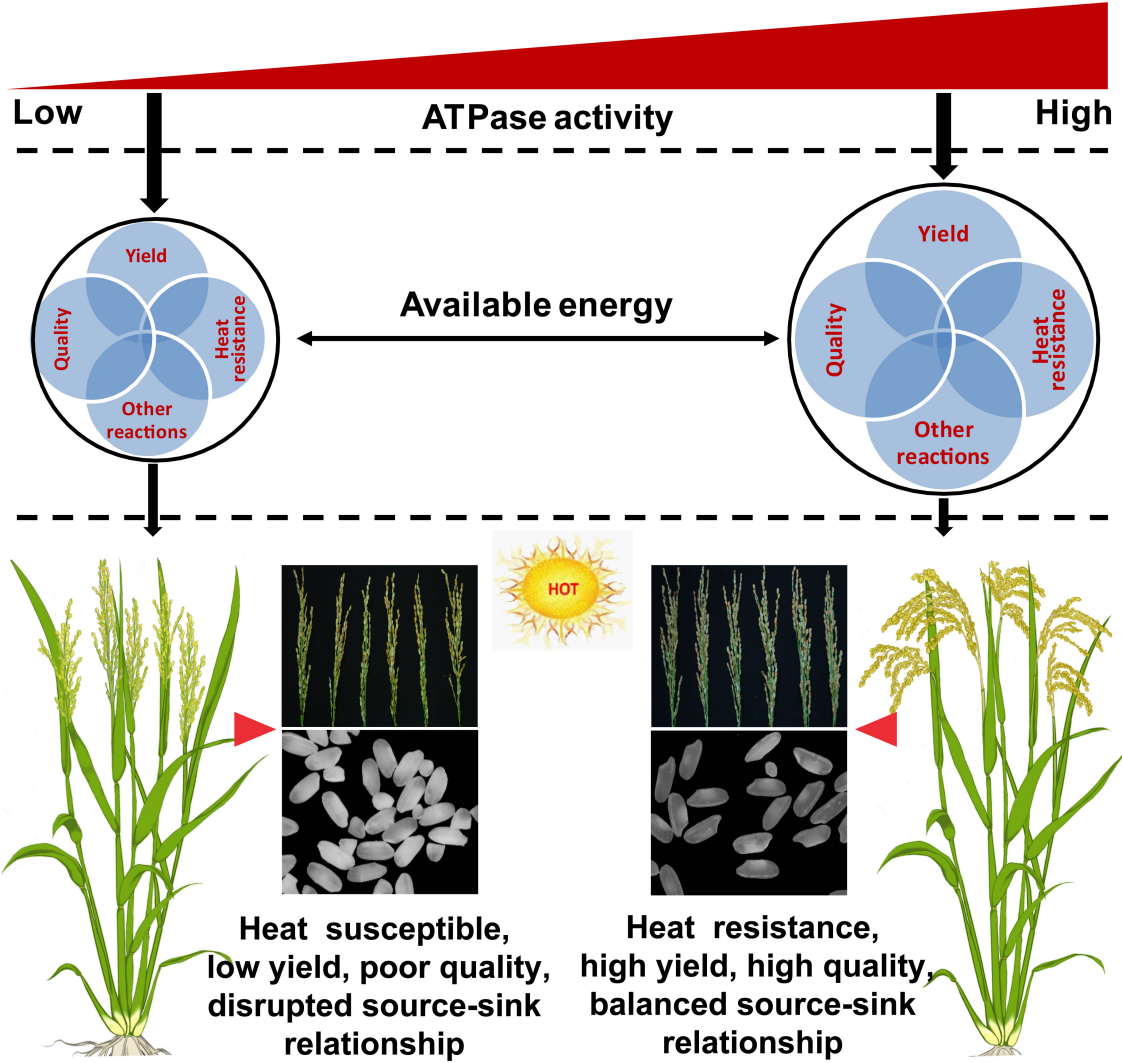
Model of energy status determined by ATPase functioned in heat resistance, yield and quality of rice under moderate hot conditions. Under moderate hot condition, heat resistance, high yield and good quality were showed in ZLY30, while heat susceptible, low yield and poor quality were showed in LLY35. This was mainly ascribed to the higher available energy determined by ATPase activity, rather than the ATP deficit, as higher ATP content were showed in LLY35 than ZLY30. Higher ATPase activity could enlarge the available energy to provide sufficient energy for the formations of heat response, yield and quality, which were considered as high energy cost processes. In contrast, lower ATPase activity could limit the available energy resulting in heat susceptible, low yield and poor quality. Furthermore, energy deficit could block the sucrose unloading in grains, which disorder the source-sink relationship. This in turns exacerbated energy shortage. The black circle means the available energy determined by ATPase. The four grayish blue circles mean the processes of heat resistance, yield, quality and other reactions including sucrose transportation, nitrogen metabolism, and protein turnovers.

Energy metabolism plays an important role in plant growth and development during the entire life cycle (De Block et al., 2005; Baena-González et al., 2007; Baena-González and Sheen, 2008; Chin et al., 2014). As traits such as high yield, high quality, and heat resistance require large amounts of energy (De Block and Van Lijsebettens, 2011), the energy status of ZLY30 is likely higher than that of LLY35. However, the ATP content was higher in LLY35 than in ZLY30 (**Figure 5**). This was inconsistent with the results of previous studies showing that the ATP content was higher in stress-resistant cultivars than in stress-susceptible cultivars (De Block et al., 2005; Jiang et al., 2020; Li et al., 2020d). However, the present findings have been reported in rice seedlings subjected to cold stress (Yu et al., 2020). ATP needs to be hydrolyzed by ATPase before it can be utilized by plants; thus, the energy status (available energy) might be determined by ATPase activity rather than ATP itself (Lin and Hardie, 2018). In this case, the higher ATPase activity observed in ZLY30 compared with LLY35 could provide sufficient energy for supporting the heat response, as well as high yield and quality (**Figure 5**). Therefore, higher ATPase activity under certain circumstances can improve the energy status in plants and meet the energy demand required for the processes underlying heat resistance, high yield, high quality, and other reactions (**Figure 7**). By contrast, low ATPase activity and insufficient available energy result in heat-susceptible, low-yield, and poor-quality plants (**Figure 7**).

Communication between source organs and sink organs plays a key role in the assimilation and partitioning of carbohydrates during plant growth and development, including the formation of yield and quality as well as the response to stress (Yu et al., 2015; Chen et al., 2019). These relationships are regulated by a complex signaling network involving sugars, hormones, and environmental factors (Hastilestari et al., 2018; Rodrigues et al., 2019; Lu et al., 2020). The possibility that these source–sink relationships are regulated by the energy status has not yet been documented (De Block and Van Lijsebettens, 2011; Yu et al., 2015). Dry matter weight and carbohydrate accumulation and allocation, as well as the expression of the sucrose transporter genes *SUT1* and *SUT2*, were higher in ZLY30 than in LLY35 under different environmental conditions (**Figures 2**-**4**). This balanced source–sink relationship in ZLY30 might be attributed to its high energy status stemming from its higher ATPase activity (**Figure 5**). Sucrose unloading in the grains *via* an apoplast pathway (active transport) uses stored energy to move particles against their concentration gradients (Foster and Miklavcic, 2020; Munns et al., 2020). Large amounts of energy are consumed in this process. A low energy status might disturb source–sink relationships and inhibit energy metabolism in grains (**Figure 7**). However, additional studies are needed to clarify the mechanism underlying the role of energy status in sucrose transport in source and sink organs, especially under limited energy conditions.

### Function of SnRK1 and TOR in energy allocation in grains

TOR and SnRK1 are two energy-sensing kinases that act in opposite directions in the regulation of metabolic-driven processes (Dröge-Laser and Weiste, 2018; Margalha et al., 2019; Fichtner et al., 2021; Li et al., 2021b). However, the activity of these kinases in the two cultivars showed similar patterns of variation under different environmental conditions (**Figure 6**). In some cases, the activity of the TOR and SnRK1 kinases only increased slightly, and they did not always act antagonistically (Rodriguez et al., 2019; Yu et al., 2020). SnRK1 can trigger extensive transcriptional changes to restore homeostasis and promote cell survival by sensing energy deficits (Baena-González and Sheen, 2008), and TOR can maintain a high rate of ribosome biogenesis, translation initiation, and nutrient import and thus promote plant growth when energy is sufficient; both TOR and SnRK1 can also be induced to confer resistance to stress in plants (Rodrigues et al., 2019). However, the relative expression levels of *SnRK1* and *TOR* were higher in LLY35 than in ZLY30 (**Figure 6**). This indicates that SnRK1, rather than TOR, was the main contributor to yield and quality formation of the two cultivars by inhibiting ATPase activity, which resulted in the low energy status observed in LLY35 (**Figures 6**, **7**). SnRK1 can be activated by a decrease in energy level, which is triggered by an increase in the AMP/ATP ratio detected by AMPK or by sugar starvation (Robaglia et al., 2012); however, inhibition of ATPase by SnRK1 has not yet been documented and requires further study.

## Conclusion

No significant relationships were observed among the HSI, yield, and quality in 49 early rice cultivars under normal temperature conditions. One heat-resistant cultivar with high yield and quality (ZLY30) and a heat-susceptible cultivar with low yield and poor quality (LLY35) were selected and planted under different environmental conditions. Yield was higher and CD was lower in ZLY30 than in LLY35 across all sowing times. The decreases in yield and increases in the CD at higher temperatures were more pronounced in LLY35 than in ZLY30. The accumulation and allocation of dry matter weight and NSC were higher in ZLY30 than in LLY35. Similar patterns were observed in the relative expression levels of *SUT1* and *SUT2* in grains, which were mainly responsible for sucrose unloading in grains. The ATP content was higher in the grains of LLY35 than in the grains of ZZLY30, but ATPase activity was lower in the former than in the latter, which might be regulated by SnRK1. Therefore, the higher ATPase activity in ZLY30 was the main factor responsible for its improved energy status, which resulted in heat resistance, high yield, and high quality; the lower ATPase activity in LLY35 resulted in heat susceptibility, low yield, and poor quality.

## Data availability statement

The original contributions presented in the study are included in the article/**Supplementary Material**. Further inquiries can be directed to the corresponding authors.

## Author contributions

GF, LT and ZW conceived the original research plan and supervised the research work. TC, JM, CX, NJ, and GL performed research. WF, BF, and DW analyzed the data. TC and GF wrote the paper. All authors contributed to the article and approved the submitted version.
