# Supplementary material for: Increased ATPase activity promotes heat-resistance, high-yield, and high-quality traits in rice by improving energy status
Source: Front Plant Sci. 2022 Dec 19;13:1035027. doi: 10.3389/fpls.2022.1035027 (PMC9806274; doi:10.3389/fpls.2022.1035027)
Supplement: Supplementary file 1 [file DataSheet_1.docx]

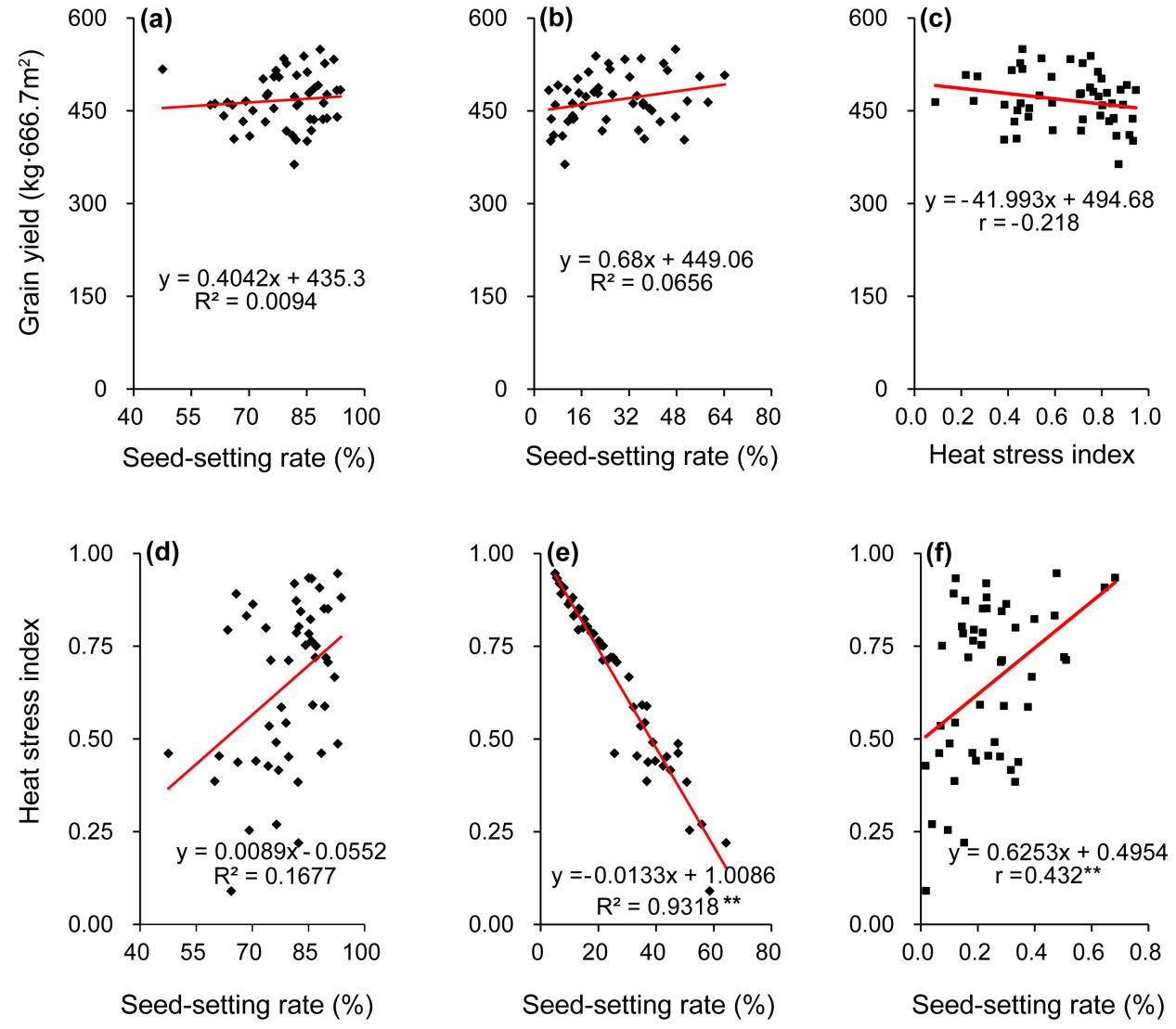


**FIGURE S1** Correlations showed among the seed-setting rate, grain yield and heat stress index. **a,** the correction between the grain yield and seed-setting rate under control; **b,** the correction between the grain yield and seed-setting rate under heat stress; **c,** the correction between the grain yield and heat stress index; **d,** the correction between the heat stress index and seed-setting rate under control; **e,** the correction between the heat stress index and seed-setting rate under heat stress; **f,** the correction in heat stress index between the treatments of 36℃ and 38℃.

**Table S1 Primer sequences used in qRT-PCR.**

| **Gene** | **Forward (5’-3’)** | **Reverse (5’-3’)** |
| --- | --- | --- |
| *SnRK1a* | ACAACCAGTGGCTACCTTGG | CGATGATCAGTGGCTGAGTT |
| *SnRK1b* | ATATCAGGCGCCGAATACTG | TGTGCCTGAAGAACTTGCTG |
| *SUT1* | GGTGGCATGCTGCTATTGTA | GTTAGCTGTGCCAGGTCCAT |
| *SUT2* | CCGTTCACCGTTACTCCATC | GAGGCTCTTGCACTGATGCT |
| *TOR* | GCTGAACGCTGCAATGACTA | ACCGAACAAGTACTGGAGCA |

**Table S2 The early indica rice varieties with different heat resistance and yields.**

| Cultivars | Seed-setting rate (%) | | Heat stress index | Grain yield  （kg/666.7m^2^） |
| --- | --- | --- | --- | --- |
|  | **Control** | **Heat stress** |  |  |
| Jinyou458 | 77.0±7.5 | 45.0±21.3 | 0.416 | 515.4±53.4 |
| Liangyouzao17 | 79.7±5.5 | 43.7±7.0 | 0.452 | 526.9±32.4 |
| Lingliangyou211 | 69.2±8.5 | 51.6±7.8 | 0.254 | 465.8±23.9 |
| Lingliangyou942 | 64.4±6.2 | 58.6±7.2 | 0.090 | 463.9±24.6 |
| Rongyou585 | 82.4±10.2 | 64.3±8.0 | 0.220 | 507.6±17.6 |
| Zhuliangyou609 | 60.0±6.0 | 36.8±8.6 | 0.386 | 459.6±66.2 |
| Zhuliangyou30 | 76.4±6.9 | 38.9±7.0 | 0.491 | 454.0±21.8 |
| YouI336 | 76.5±7.8 | 55.9±5.3 | 0.270 | 505.5±46.7 |
| Yuliangyou4156 | 88.5±7.0 | 47.6±4.7 | 0.462 | 549.5±33.0 |
| Zhuliangyou829 | 61.2±6.5 | 33.4±5.5 | 0.454 | 461.9±9.10 |
| Ganzaoxian51 | 81.8±3.3 | 10.4±1.9 | 0.873 | 363.6±21.8 |
| Lingliangyou611 | 70.2±9.6 | 9.6±1.4 | 0.863 | 409.2±43.6 |
| Luliangyou35 | 81.3±10.5 | 6.6±2.1 | 0.919 | 410.6±25.0 |
| Zhongzao35 | 79.7±5.2 | 23.0±5.1 | 0.712 | 417.9±64.5 |
| Xiangzaoxian6 | 85.1±4.7 | 5.6±1.9 | 0.934 | 401.0±30.6 |
| Xiangzaoxian32 | 86.9±6.8 | 24.3±2.7 | 0.720 | 435.9±28.2 |
| Xiangzaoxian42 | 90.2±4.6 | 13.4±3.5 | 0.851 | 438.1±61.5 |
| Lingliangyou7717 | 89.2±11.5 | 13.2±2.8 | 0.852 | 436.6±18.7 |
| Zhongzao39 | 85.9±4.6 | 5.8±1.9 | 0.933 | 436.7±12.5 |
| Rongyou286 | 87.6±5.8 | 25.6±3.4 | 0.746 | 417.5±15.4 |

**Table S3 The quality showed in the cultivars with different heat resistance and yields.**

| Cultivars | BRR | HRR | MRR | CD | Translucency | GL | AR | ASV | GC | AC | BRPC |
| --- | --- | --- | --- | --- | --- | --- | --- | --- | --- | --- | --- |
| Jinyou 458 | 64.2±0.17 | 35.4±0.06 | 53.9±0.22 | 6.7±0.43 | 4 | 6.2±0.02 | 2.5 | 5.7±0.26 | 76±0.72 | 21.0±0.17 | 9.00±0.56 |
| Liangyouzao 17 | 81.3±0.20 | 34.5±0.10 | 68.8±0.03 | 4.0±0.22 | 2 | 7.0±0.09 | 3.1 | 4.8±0.34 | 74±0.66 | 20.0±0.22 | 9.41±0.49 |
| Lingliangyou 211 | 80.0±0.26 | 46.3±0.20 | 70.6±0.07 | 3.0±0.13 | 2 | 6.7±0.05 | 3.0 | 5.8±0.42 | 74±0.64 | 13.6±0.28 | 9.05±0.61 |
| Liangliangyou 942 | 80.0±0.36 | 37.7±0.25 | 68.3±0.15 | 2.6±0.80 | 3 | 6.7±0.02 | 2.9 | 5.2±0.61 | 81±0.59 | 23.1±0.31 | 8.69±0.23 |
| Rongyou585 | 80.7±0.21 | 43.7±0.06 | 69.8±0.20 | 1.6±0.11 | 2 | 6.5±0.01 | 2.7 | 6.0±0.57 | 64±0.76 | 22.0±0.46 | 8.70±0.24 |
| Yiliangyou 4156 | 70.2±0.32 | 43.7±1.08 | 59.9±0.47 | 3.2±0.42 | 2 | 6.5±0.03 | 2.9 | 6.0±0.53 | 80±1.42 | 26.3±0.52 | 9.82±0.52 |
| Zhuliangyou 30 | 81.2±0.30 | 38.2±0.25 | 67.6±0.25 | 1.6±0.25 | 3 | 6.5±0.06 | 2.7 | 5.5±0.44 | 76±0.98 | 20.7±0.43 | 9.24±0.64 |
| You I336 | 81.6±0.25 | 44.5±0.15 | 68.6±0.06 | 3.2±0.21 | 2 | 6.5±0.01 | 2.7 | 5.2±0.55 | 76±0.85 | 22.0±0.51 | 9.64±0.23 |
| Zhuliangyou 609 | 80.1±0.20 | 51.1±0.35 | 65.9±0.21 | 3.4±0.28 | 4 | 6.4±0.02 | 2.5 | 5.7±0.26 | 61±0.63 | 25.8±0.24 | 9.25±0.35 |
| Zhuliangyou 829 | 68.8±0.35 | 30.4±0.20 | 54.9±0.36 | 7.9±1.25 | 3 | 7.0±0.05 | 2.9 | 5.5±0.38 | 82±0.54 | 26.2±0.33 | 9.53±0.57 |
| Average | 76.8±6.46 | 40.6±6.34 | 64.8±6.24 | 3.7±2.05 | 2.7±0.82 | 6.6±0.25 | 2.8±0.20 | 5.5±0.38 | 74.4±6.90 | 22.1±3.79 | 9.2±0.38 |
| CV | 0.084 | 0.156 | 0.096 | 0.552 | 0.305 | 0.038 | 0.073 | 0.069 | 0.093 | 0.172 | 0.041 |
| Ganzaoxian 51 | 78.5±0.12 | 54.9±0.08 | 66.9±0.42 | 4.4±0.25 | 2 | 6.5±0.02 | 3.1 | 6.5±0.41 | 51±0.46 | 18.3±0.58 | 9.94±0.35 |
| Rongyou 286 | 69.0±0.21 | 35.3±0.26 | 59.2±0.26 | 4.0±0.33 | 2 | 6.9±0.04 | 3.0 | 5.5±0.56 | 68±0.81 | 21.3±0.56 | 9.27±0.62 |
| Lingliangyou 611 | 60.4±0.15 | 28.2±0.08 | 50.8±0.07 | 2.2±0.16 | 3 | 6.4±0.01 | 2.9 | 4.3±0.47 | 74±0.66 | 14.6±0.39 | 9.26±0.51 |
| Luliangyou 35 | 70.3±0.25 | 36.6±0.11 | 58.7±0.09 | 5.9±0.87 | 4 | 6.1±0.03 | 2.4 | 5.0±0.30 | 75±0.59 | 20.0±0.66 | 9.88±0.62 |
| Zhongzao 35 | 79.9±0.17 | 42.2±0.09 | 68.3±0.11 | 6.5±0.69 | 4 | 6.3±0.02 | 2.6 | 5.2±0.26 | 76±0.95 | 19.2±0.57 | 8.37±0.47 |
| Xiangzaoxian 6 | 77.4±0.12 | 58.5±0.42 | 67.2±0.10 | 3.9±0.43 | 3 | 5.2±0.04 | 2.0 | 6.0±0.41 | 55±0.87 | 24.3±0.24 | 11.80±0.98 |
| Xiangzaoxian 32 | 79.8±0.20 | 61.6±0.15 | 69.2±0.15 | 5.2±0.39 | 3 | 5.4±0.03 | 2.1 | 5.5±0.20 | 60±1.02 | 22.6±0.37 | 10.80±0.67 |
| Xiangzaoxian 42 | 79.9±0.10 | 60.5±0.03 | 69.1±0.26 | 1.9±0.21 | 2 | 6.6±0.05 | 3.0 | 6.0±0.33 | 70±1.56 | 15.5±0.46 | 9.47±0.64 |
| Lingliangyou 7717 | 80.8±0.25 | 42.8±0.06 | 68.7±0.09 | 3.3±0.17 | 4 | 6.0±0.02 | 2.4 | 4.5±0.58 | 65±0.61 | 20.5±0.75 | 8.50±0.51 |
| Zhongzao 39 | 80.0±0.06 | 52.5±0.08 | 67.5±0.08 | 5.9±0.82 | 4 | 5.8±0.04 | 2.1 | 5.2±0.44 | 73±0.88 | 25.1±0.65 | 8.91±0.63 |
| Average | 75.6±6.78 | 47.3±11.82 | 64.6±6.20 | 4.3±1.57 | 3.1±0.88 | 6.1±0.53 | 2.6±0.42 | 5.4±0.68 | 66.7±8.77 | 20.1±3.43 | 9.6±1.05 |
| CV | 0.090 | 0.250 | 0.096 | 0.364 | 0.282 | 0.087 | 0.164 | 0.127 | 0.131 | 0.170 | 0.109 |

*Note:* BRR, Brown rice rate; HRR, Head rice rate; MRR, Milled Rice Ratio; CD, Chalkiness degree; GL, Grain length; AR, Aspect ratio; ASV, alkali spreading value; GC, Gel consistency; AC, amylose content; BRPC, protein content.

**Table S4 Correlation analysis of rice quality with heat stress index and grain yields.**

| Quality parameters | Heat stress  index of 36℃ | Heat stress  index of 38℃ | Grain yield |
| --- | --- | --- | --- |
| BRR | -0.072 | -0.168 | 0.167 |
| HRR | 0.088 | 0.242 | -0.151 |
| MRR | -0.067 | -0.133 | 0.098 |
| CD | 0.505* | 0.221 | -0.159 |
| Translucency | 0.458* | 0.275 | -0.038 |
| GL | -0.392 | -0.394 | 0.236 |
| AR | -0.440 | -0.245 | 0.026 |
| ASV | -0.058 | -0.201 | 0.002 |
| GC | -0.068 | -0.423 | 0.211 |
| AC | 0.068 | -0.271 | 0.305 |
| BRPC | 0.098 | 0.179 | -0.146 |

*Note:* BRR, Brown rice rate; HRR, Head rice rate; MRR, Milled Rice Ratio; CD, Chalkiness degree; GL, Grain length; AR, Aspect ratio; ASV, alkali spreading value; GC, Gel consistency; AC, amylose content; BRPC, protein content.
